# Controllable Phase Transformation and Mid-infrared Emission from Er^3+^-Doped Hexagonal-/Cubic-NaYF_4_ Nanocrystals

**DOI:** 10.1038/srep29871

**Published:** 2016-07-25

**Authors:** Dandan Yang, Dongdan Chen, Huilin He, Qiwen Pan, Quanlan Xiao, Jianrong Qiu, Guoping Dong

**Affiliations:** 1 State Key Laboratory of Luminescent Materials and Devices and Institute of Optical Communication Materials, School of Materials Science and Engineering, South China University of Technology, Guangzhou 510640, China; 2 SZU-NUS Collaborative Innovation Center for Optoelectronic Science & Technology, Key Laboratory of Optoelectronic Devices and Systems of Ministry of Education and Guangdong Province, College of Optoelectronic Engineering, Shenzhen University, Shenzhen 518060, China

## Abstract

The morphology of hexagonal phase NaYF_4_:Er^3+^ nanorods synthesized by hydrothermal method changed greatly after a continuing calcination, along with a phase transformation to cubic phase. Photoluminescence (PL) spectra indicated that mid-infrared (MIR) emission was obtained in both hexagonal and cubic phase NaYF_4_:Er^3+^ nanocrystals for the first time. And the MIR emission of NaYF_4_:Er^3+^ nanocrystals enhanced remarkably at higher calcination temperature. To prevent uncontrollable morphology from phase transformation, the cubic phase NaYF_4_:Er^3+^ nanospheres with an average size of ~100 nm were prepared *via* a co-precipitation method directly. In contrast, the results showed better morphology and size of cubic phase NaYF_4_:Er^3+^ nanocrystals have realized when calcined at different temperatures. And PL spectra demonstrated a more intense MIR emission in the cubic phase NaYF_4_:Er^3+^ nanocrystals with an increasing temperature. Besides, the MIR emission peak of Er^3+^ ions had an obvious splitting in cubic phase NaYF_4_. Therefore, cubic phase NaYF_4_:Er^3+^ nanospheres with more excellent MIR luminescent properties seems to provide a new material for nanocrystal-glass composites, which is expected to open a broad new field for the realization of MIR lasers gain medium.

Mid-infrared (MIR) fiber lasers operating at ~3 μm are in great demand for a variety of applications including medical surgery, hazardous chemical detection, remote sensing, eye safe laser radar and atmosphere pollution monitoring^1^,^2^,^3^,^4^,^5^. This mainly relies on the two unique features of this wavelength range: (1) well-known atmospheric transparency windows are located in this wavelength range; (2) various molecules have strong rovibronic absorption lines in this ‘molecular fingerprint’ region of the electromagnetic spectrum^1^. So far, glass-based materials (conventional glass and glass ceramics materials) may be the most important material used for optical fiber production^6^,^7^,^8^. However, MIR emission at ~3 μm is the closely spaced energy levels transition, which is liable to be quenched by multiphonon non-radiative decay, hence, it seems difficult to obtain intense MIR emission in most glass-based materials. In comparison with glass-based materials, nanocrystal (NC) materials are thought to be more suitable as MIR laser host because of their many advantages with smaller particle size, higher chemical stability, more nature synthesis method, lower thermal lens effect and higher doping concentration of trivalent rare-earth (RE) ions^9^,^10^,^11^,^12^,^13^. Thus, in our previous work^11^, a novel co-melting technology has been proposed to introduce RE ions doped NC into glass matrixes to form NC-doped glass composite (NGC). The NGC can provide a stable crystalline field for active ion to realize higher MIR luminescent efficiency, and have potential for fiber-drawing, which will open a brand new field for the realization of MIR fiber lasers.

NGC and co-melting technology present a new approach to research the MIR composite optical fiber preforms and fiber forming. However, the preparation of NGC brings forward rigorous demands to the MIR emission NCs, such as high MIR luminescent intensity, controllable morphology and better thermal stability etc. So it is extremely important to search suitable MIR emission NCs for NGC preparation. As an ideal host, fluorides with low phonon frequency and intense crystalline field, have been widely studied^14^,^15^,^16^. Among these RE fluorides, NaYF_4_ with low vibration energy (<400 cm^−1^)^17^, controllable morphology and particle size^18^ have attracted widespread attention to study the visible up-conversion and near-infrared emission of RE ions in NaYF_4_ NCs^17^,^18^,^19^,^20^,^21^. To our best knowledge, no any works are reported about the MIR emission in RE-doped NaYF_4_ NCs yet. In consideration of the above superiorities, NaYF_4_ NCs are thought to be one of the best MIR luminescent host and chosen as the host material in this work. There are two kinds of NaYF_4_ host material: cubic (α) and hexagonal (β) phases. At present, a number of methods have been available to synthesize these two kinds of NaYF_4_ NCs, including solid treatment, co-precipitation method, hydrothermal/solvothermal synthesis and the surfactant-controlled organometallic pyrolysis approach^17^,^18^,^19^. In this work, environment friendly hydrothermal and co-precipitation methods are chosen to prepare the two kinds of NaYF_4_ host material, respectively.

Up to now, numerous RE ions in suitable host have realized the MIR emission, such as Er^3+^, Ho^3+^ and Dy^3+ ^22,23,24. Among them, Er^3+^ emission at 2.7 μm attributed to the ^*2*^*I*_*11/2*_ → ^*4*^*I*_*13/2*_transition, plays an important role in the investigations and has been achieved in many kinds of glasses^25^,^26^, glass ceramics^27^,^28^ and single crystals^29^. Hence, in this paper, utilizing Er^3+^ ions as active centre, Er^3+^-doped NaYF_4_ NCs with intense MIR fluorescence was investigated, which is expected to be used as gain medium of MIR fiber laser.

## Results

### Structures, morphologies and fluorescence of β-NaYF_4_:5%Er^3+^ NCs

**Figure 1:**
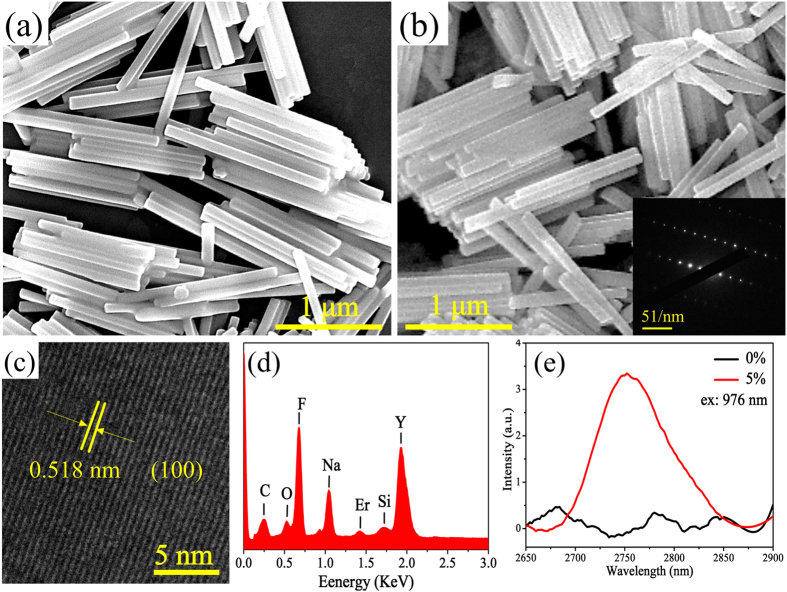
(**a**,**b**) SEM images of the as-prepared β-NaYF_4_ and β-NaYF_4_:5%Er^3+^ nanocrystals. (**c**) HRTEM image and (**d**) EDS spectrum of as-prepared β-NaYF_4_:5%Er^3+^ nanocrystals. (**e**) The MIR emission spectra of as-prepared β-NaYF_4_ and β-NaYF_4_:5%Er^3+^ nanocrystals. The inset in (**b**) is corresponding SAED pattern.

Using hydrothermal synthesis, pure hexagonal phase NaYF_4_ NCs was fabricated, as shown in XRD patterns in Supplementary Fig. S1. After the substitution of Y^3+^ ions by 5% Er^3+^ ions, there is no influence on crystal structure. Examining the XRD patterns carefully, it can be noted that (110) crystal facets has higher diffraction intensity than the (101) crystal facets in as-prepared NCs, indicating anisotropic growth along *c*-axis during the synthesis process, which can be proved by the SEM results. It can be seen in Fig. 1(a), all of the as-prepared β-NaYF_4_ samples are well-dispersed nanorods with ~100 nm in width as well as ~1.3 μm in length. The morphology remains monodisperse nanorods with the similar size after the doping of Er^3+^ ions (Fig. 1(b)). The insert in Fig. 1(b) reveals the selected area electron diffraction (SAED) pattern of a single β-NaYF_4_:5%Er^3+^ nanorod, which indicates the single β-NaYF_4_:5%Er^3+^ nanorod exhibits a single crystalline character. In HRTEM image of a single β-NaYF_4_:5%Er^3+^ nanorod (Fig. 1(c)), a series of crystal facets with the spacing value of ~0.518 nm is observed, which corresponds to the (100) crystal facet of β-NaYF_4_ crystals. Therefore, it can be deduced that the addition of Er^3+^ ions has no obvious effect on the phase, size and morphology of β-NaYF_4_ NCs. Fig. 1(d) describes the EDS spectrum of β-NaYF_4_:5%Er^3+^ NCs. The peaks assigned to designed elements (i.e. Na, Y, F and Er) are clearly observed in this picture. Besides, the O, C and Si elements were introduced during the synthesis and measurement process. It is believed that the β-NaYF_4_:5%Er^3+^ NCs are mainly consisted of Na, Y, F and Er elements.

The MIR emission spectrum of Er^3+^ ions doped β-NaYF_4_ NCs is shown in Fig. 1(e). Under 976 nm laser diode (LD) pumping, an obvious MIR emission peak at ~2.75 μm attributed to the Er^3+^: ^4^I_11/2_ → ^4^I_13/2_ transition is observed in β-NaYF_4_ NCs. However, the existence of organic ligands such as OH^−^ etc. (Supplementary Fig. S6), which have a strong absorption at around ~3 μm, can greatly reduce the MIR emission intensity of Er^3+^ ions. Therefore, in this study, a convenient method, heat treatment at different temperatures in air atmosphere, was chosen to remove the organic ligands. This is because at high temperature, the organic ligands would volatilize, and F^−^ ions also have hydroscopic property.

### Thermal treatment of β-NaYF_4_:5%Er^3+^ NCs

**Figure 2:**
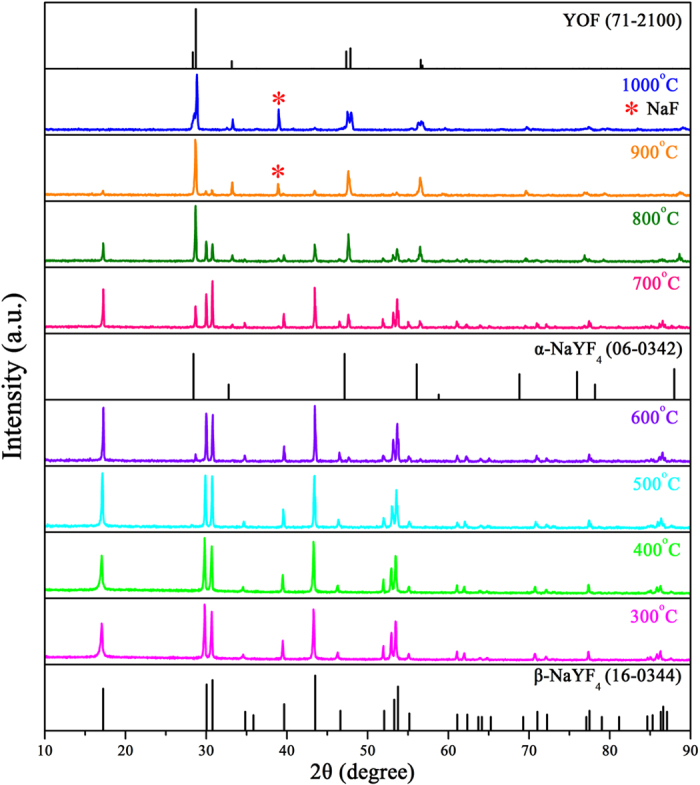
XRD patterns of β-NaYF_4_:5%Er^3+^ nanocrystals calcined at different temperatures for 2 h.

In order to remove organic ligands and realize intense MIR emission, the β-NaYF_4_:5%Er^3+^ NCs were calcined at different temperatures for 2 h. The β-NaYF_4_:Er^3+^ NCs calcined at series of temperatures are labeled as B-NYF, and B-NYF-X (X = 300, 400, …, 1000) represents the B-NYF of corresponding temperature. The crystal structures of B-NYF are displayed in Fig. 2. As shown in this pattern, samples remain pure hexagonal phase less than 600 °C. At 600 °C, an obvious phase transformation from hexagonal to cubic has occurred. When the temperature reaches 900 °C, nearly all β-crystals transform into α-crystals, while the residual β-crystals could be neglected. Except for NaYF_4_, a small number of NaF (JCPDF: 36–1455) is detected at this temperature along with the diffraction peaks shift slightly to higher 2θ region. Further increase of temperature results in the occurrence of rhombohedral phase YOF (JCPDF: 71–2100), which is due to the fact that oxygen atoms in the environment obtain energy entering into crystal lattice to replace fluorine atoms. Thus, the increase of temperature from 300 °C to 1000 °C leads to two phase transformation processes, one is from β-NaYF_4_ to α-NaYF_4_, and the other is the oxidation from α-NaYF_4_ to YOF. Moreover, it is noteworthy that the diffraction peaks of all samples have narrow full width at half maximum (FWHM), indicating the growth of crystalline particles has better crystallinity, which is benefit for fluorescence emission.

It has been reported that the unique Raman peaks can be efficiently used to distinguish cubic and hexagonal phases of NaYF_4_ 30. So Raman analysis (Supplementary Fig. S2) was used to further prove the XRD results. When calcined less than 600 °C, only dominant Raman peaks of β-NaYF_4_:5%Er^3+^ NCs are detected^31^,^32^,^33^. At 600 °C, two other weak Raman peaks which attributed to the vibrations from the α-NaYF_4_ appear^30^. With the increase of temperature, the peaks of β-NaYF_4_ get weak, and the peaks of α-NaYF_4_ become strong. Until 900 °C, the peaks of hexagonal phase can be ignored comparing with cubic phase. At same time, three new Raman peaks are observed, owing to the vibrations from YOF^34^ and NaF. When the temperature reaches 1000 °C, there are only Raman peaks of YOF, NaF and α-NaYF_4_ observed, without peaks of β-NaYF_4_. Above results match well with the XRD patterns, indicating the occurrence of two phase transformation during the calcination process of β-NaYF_4_:5%Er^3+^ NCs. According to the thermogravimetry-differential scanning calorimtry (TG-DSC) curves (Supplementary Fig. S3), these two phase transformation processes occur at 698.9 and 984 °C, respectively. Due to the fast heating rate and short holding time during the TG-DSC measurement, a slight difference is found between TG-DSC curves and XRD patterns.

**Figure 3:**
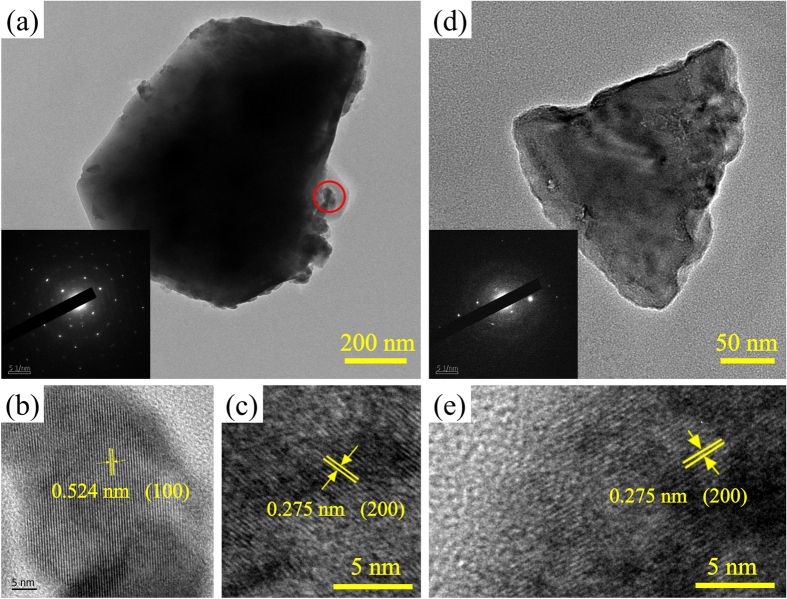
(**a**) TEM image of β-NaYF_4_:5%Er^3+^ nanocrystals calcined at 700 °C for 2 h. (**b**,**c**) HRTEM images of the little particle in red circle and the micro blocks in (**a**), respectively. (**d**) TEM image of α-NaYF_4_:5%Er^3+^ nanocrystals calcined at 700 °C for 2 h. (**e**) HRTEM image of samples in (**d**). The inserts in (**a**,**d**) are corresponding SAED pattern.

As displayed in the SEM images of β-NaYF_4_:5%Er^3+^ NCs calcined at different temperatures (Supplementary Fig. S4), during the process of temperature rise, the morphology has an obvious change, along with crystalline phase transformation from pure β-NaYF_4_ to α-NaYF_4_. The detailed morphology and structure of representative B-NYF-700 samples are shown in Fig. 3(a–c). From Fig. 3(a), it can be seen that the B-NYF-700 samples consist of two kinds of morphologies, micro blocks and little irregular particles. Their corresponding HRTEM images indicate the irregular particles are β-NaYF_4_ crystals, and the micro blocks are α-NaYF_4_ crystals. This is because crystal lattice fringe with spacing *d* value of ~0.524 nm in Fig. 3(b) corresponds to the (100) crystal facets of β-NaYF_4_, and ~0.275 nm in Fig. 3(c) matches with the (200) crystal facets spacing of α-NaYF_4_. As a result, the corresponding SAED pattern reveals polycrystalline character. For B-NYF-1000 samples, there are mainly three kinds of morphologies observed in Supplementary Fig. S5(a,b): nanoplates, irregular nanoparticles and micro blocks. In Supplementary Fig. S5(c), the crystal facets spacing of ~0.265 nm corresponds to (111) crystal facets of NaF. And the EDS spectrum (Supplementary Fig. S5(g)) illustrates the nanoplates mainly consist of Na and F elements. So it can be inferred the thin nanoplates are NaF plates. In Supplementary Fig. S5(d,e), the ~0.318 nm and ~0.328 nm are the crystal lattice fringes of (006) and (101) crystal facets in YOF crystal, which reveals these two kinds of particles are YOF crystals. Therefore, B-NYF-1000 samples consist of three morphologies: NaF nanoplates, YOF irregular nanoparticles and micro blocks.

Based on above results, it can be inferred that B-NYF samples are located in stable state, and remain their original morphology and crystal structure at low temperature (<600 °C). When calcined at 500 °C, β-NaYF_4_ NCs obtain energy and start to fuse slightly. But this temperature is too low to provide enough energy for the phase transformation from hexagonal to cubic phase. Once rising temperature to 600 °C, the energy is enough to fuse β-NaYF_4_ NCs thoroughly and form α-NaYF_4_ crystals. At higher temperature, α-NaYF_4_ crystals as high temperature phase gain more energy to nucleate and growth in quantity. Nevertheless, the absence of shaping and stabilizing agents leads to the uncontrollable growth of α-NaYF_4_ crystal nucleus. When the temperature reaches to 1000 °C, the energy is too high so that oxygen atoms in air obtain energy to entire into the crystal lattice and replace fluorine atoms. Finally, α-NaYF_4_ crystal transforms into YOF phase. As for the replaced fluorine atoms, some of them are volatilized, and the remainders combine with sodium atoms to form NaF.

**Figure 4:**
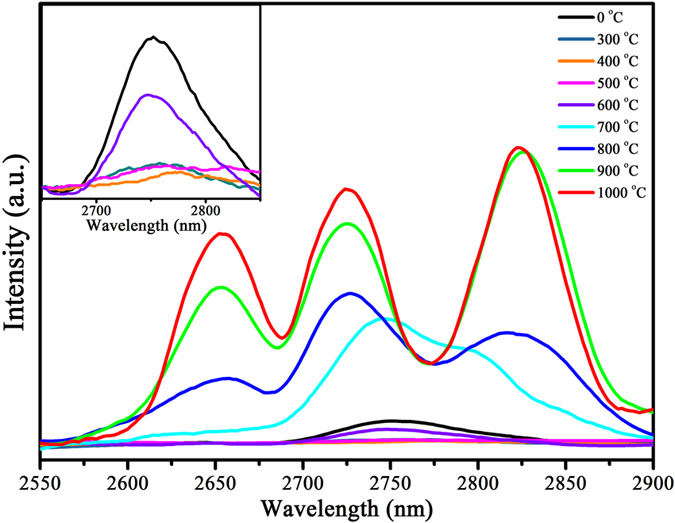
MIR emission spectra of β-NaYF_4_:5%Er^3+^ nanocrystals calcined at different temperatures for 2 h. Insert: the enlarged image of the curves of β-NaYF_4_:5%Er^3+^ nanocrystals (calcined from 0 to 600 °C for 2 h) in the range from 2650 nm to 2850 nm, respectively. The 0 °C stands for as-prepared β-NaYF_4_:5%Er^3+^ nanocrystals.

The MIR emission spectra of β-NaYF_4_:5%Er^3+^ NCs calcined at different temperatures for 2 h is illustrated in Fig. 4. And the inset is enlarged image of β-NaYF_4_:5%Er^3+^ NCs calcined from 0 to 600 °C for 2 h. It is found that, at higher temperature, an intense MIR emission is detected. But, at lower temperature, B-NYF samples have weaker MIR emission than that of as-prepared samples. This is because the combustion and volatilization of organic ligands would introduce numerous defects on the surface of β-NaYF_4_:5%Er^3+^ NCs, and these defects could greatly quench ~2.7 μm fluorescence of Er^3+^ ions^35^. According to FTIR spectra (Supplementary Fig. S6) and TG-DSC curves (Supplementary Fig. S3), it can be noted that, more organic ligands combust and volatilize at 400 °C than at 300 °C, so B-NYF-400 samples contain more defects when they have similar morphology. Accordingly, weaker MIR emission was detected in B-NYF-400 samples. From 400 to 600 °C, the fusion and agglomeration of samples decrease some surface defects, as a result, an increasing MIR emission intensity was observed. From 700 °C, a noticeable enhancement of MIR emission in B-NYF samples with the increase of temperature can be ascribed into three main reasons as follows. Firstly, recrystallization from hexagonal NCs into cubic micro blocks efficiently reduces the defects, including surface defects and internal defects. The surface defects can greatly quench MIR emission, and the internal defects coming from phase transformation can transfer excitation energy to trap level by nonradiative relaxation to reduce pumping efficiency^36^. Secondly, the α-NaYF_4_ micro blocks have more excellent crystallinity at higher temperature. Thirdly, the FTIR spectra (Supplementary Fig. S6) show the organic ligands in the structure of B-NYF samples have been removed thoroughly upon 600 °C. In these spectra, it is worth mentioning that the emission peak appears splitting along with formation of cubic phase.

In conclusion, at high temperature, especially 900 °C, MIR fluorescence of Er^3+^ ions with high intensity was achieved in B-NYF samples. However, the transformation from β-NaYF_4_ NCs to irregular α-NaYF_4_ micro blocks would extremely limit their application. Hence, in order to prevent β-NaYF_4_ NCs transform into α-NaYF_4_ micro blocks at 700 °C, and avoid phase transformation (β → α) during the calcination process, α-NaYF_4_:5%Er^3+^ NCs were prepared as precursors directly, and then calcined at different temperatures for 2 h in air. It is expected that α-NaYF_4_:5%Er^3+^ NCs can remain original crystal phase and controllable morphology as well as size after calcination.

### Structures, morphologies and fluorescence of α-NaYF_4_:5%Er^3+^ NCs

**Figure 5:**
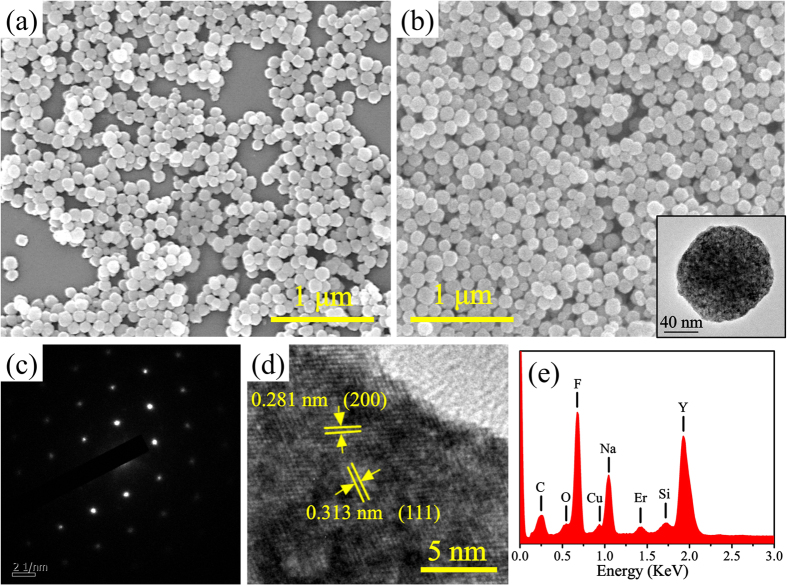
(**a**,**b**) SEM images of the as-prepared α-NaYF_4_ and α-NaYF_4_:5%Er^3+^ nanocrystals. (**c**) SAED pattern, (**d**) HRTEM image and (**e**) EDS spectrum of as-prepared α-NaYF_4_:5%Er^3+^ nanocrystals, respectively. The insert in (**b**) is TEM image of a single α-NaYF_4_:5%Er^3+^ nanocrystal.

Utilizing Na_2_EDTA as precipitator, pure cubic phase NaYF_4_:5%Er^3+^ NCs were obtained *via* co-precipitation method, as shown in XRD patterns in Supplementary Fig. S7. The morphology of as-prepared crystals is described in Fig. 5(a,b). In Fig. 5(a), the as-prepared undoped α-NaYF_4_ sample consists of a large quantity of uniform, monodisperse and spherical particles with diameter of ~100 nm. When replacing 5%Y^3+^ ions with Er^3+^ ions, there is no influence on the morphology. The insert in Fig. 5(b) is TEM image of a single α-NaYF_4_:5%Er^3+^ nanoparticle, which reveals the single α-NaYF_4_:5%Er^3+^ nanoparticle is spherical. Further crystal structure and component analysis of as-prepared α-NaYF_4_:5%Er^3+^ NCs are characterized in Fig. 5(c–e). The SAED pattern shows the single crystalline character of the individual α-NaYF_4_:5%Er^3+^ nanosphere. In the HRTEM image, the clear crystal lattice fringes with the spacing *d* values of ~0.313 nm and ~0.281 nm correspond to the (111) and (200) *d*-spacing of α-NaYF_4_. In EDS spectrum (Fig. 5(e)), except for peaks assigned to designed elements (i.e. Na, Y, F and Er) as well as the elements introduced during the synthesis and measurement process (O, C, Cu and Si), no other impurity is detected.

**Figure 6:**
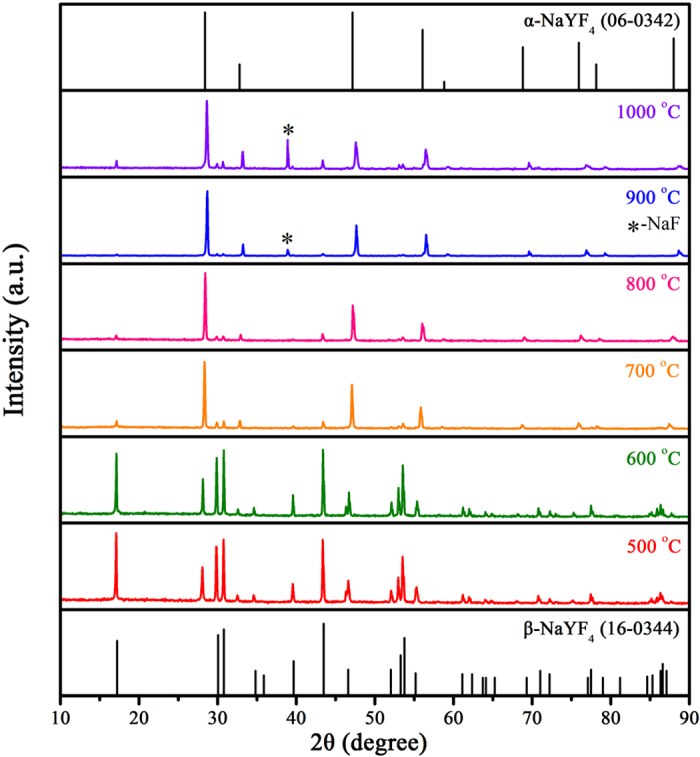
XRD patterns of α-NaYF_4_:5%Er^3+^ nanocrystals calcined at different temperatures for 2 h.

Similar to β-NaYF_4_:5%Er^3+^ NCs, as-prepared α-NaYF_4_:5%Er^3+^ NCs were calcined at different temperatures to remove organic ligands. For β-NaYF_4_:5%Er^3+^ NCs, the cubic phase appeared at 600 °C, so 500 °C was selected as beginning temperature for α-NaYF_4_:5%Er^3+^ NCs. The α-NaYF_4_:Er^3+^ NCs calcined at series of temperatures are labeled as A-NYF, and A-NYF-X (X = 500, 600, …, 1000) represents the A-NYF of corresponding temperature. Figure 6 shows XRD patterns of A-NYF. It can be noted, at 500 and 600 °C, hexagonal phase still is main phase. Herein, the temperature is hard to reach the required temperature directly during the calcination process, leading to the appearance of low temperature hexagonal phase. However, it can be observed clearly, in contrast to B-NYF samples, a considerable part of cubic phase exists in A-NYF samples at 500 and 600 °C, which indicates that a part of cubic phase NCs do not undergo the phase transformation. From 700 °C, the hexagonal phase forming during calcination process transformed into cubic phase again, and disappeared gradually with temperature rise. At 900 °C, nearly only cubic phase is monitored. As temperature reaches 1000 °C, O atoms gain energy and enter into crystal lattice, resulting in the slight shift of diffraction peaks and formation of NaF. Thus, the increase of temperature from 500 °C to 1000 °C contains two phase transformation processes: α → β at lower temperature and β → α at higher temperature. This conclusion can be proved by Raman analysis and TG-DSC curves. As shown in Raman spectra (Supplementary Fig. S8), the Raman peaks of hexagonal phase can be detected from 500 °C, and decrease gradually along with the increase of temperature. Until 900 °C, nearly only Raman peaks of cubic phase, YOF and NaF are monitored. On the basis of TG-DSC curves in Supplementary Fig. S9, three phase transformation temperatures are confirmed: cubic phase transformed into hexagonal phase at ~480 °C, then hexagonal phase transformed back cubic phase at ~692 °C, finally NaYF_4_ was oxidized into YOF upon 1000 °C.

The SEM images of A-NYF are shown in Supplementary Fig. S10. Similar with β-NaYF_4_:5%Er^3+^ NCs, the nanospheres change into bigger irregular nano-blocks after continuous calcination because of the phase transformation process. Based on the TEM results, the detailed morphology and structure of representative A-NYF samples are illustrated in Fig. 3(d). The insert is the corresponding SAED pattern, which describes the single crystalline character of the nano-blocks. The HRTEM image is displayed in Fig. 3(e), in which a crystal lattice fringe with the spacing *d* value of ~0.273 nm corresponds to the (200) crystal facet of α-NaYF_4_. Consequently, it can be deduced, when α-NaYF_4_:5%Er^3+^ NCs were calcined at different temperatures, some samples would transform into hexagonal phase at low temperature, giving rise to fusion and agglomeration of samples. From 700 °C, all samples obtain enough energy to fuse thoroughly and agglomerate to irregular nano-blocks. And just a few β-NaYF_4_ samples dispersed among the nano-blocks, which are difficult to be observed. It is important to emphasize that it is difficult to achieve completely controllable morphology for α-NaYF_4_:5%Er^3+^ NCs, but compared to B-NYF samples (Supplementary Fig. S4), A-NYF samples treated from 700 °C present pure cubic phase, smaller particle size, better uniformity and dispersibility. Accordingly, α-NaYF_4_:5%Er^3+^ NCs seem have more potential to combine with glass forming NGC.

**Figure 7:**
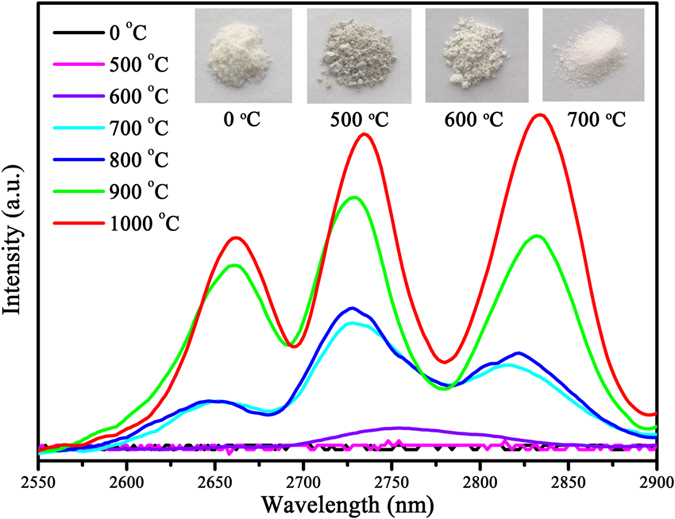
MIR emission spectra of α-NaYF_4_:5%Er^3+^ nanocrystals calcined at different temperatures for 2 h. Insert: sample photos of α-NaYF_4_:5%Er^3+^ nanocrystals calcined at different temperatures for 2 h. The 0 °C stands for as-prepared α-NaYF_4_:5%Er^3+^ nanocrystals.

MIR emission spectra of α-NaYF_4_:5%Er^3+^ NCs calcined at different temperatures for 2 h is revealed in Fig. 7. It is worth noting that, there is no MIR emission from as-prepared α-NaYF_4_:5%Er^3+^ NCs, which can be explained as follows: (1) the OH^−^ in the structure of as-prepared α-NaYF_4_:5%Er^3+^ NCs have greatly absorbed MIR emission of Er^3+^ ions (Supplementary Fig. S11); (2) according to Raman analysis (Supplementary Fig. S8), the poor crystallinity of as-prepared α-NaYF_4_:5%Er^3+^ NCs induces that there is no MIR emission of Er^3+^ ions; (3) the smaller size and bigger specific surface area of the as-prepared α-NaYF_4_:5%Er^3+^ NCs introduce more surface defects, which can quench MIR emission of Er^3+^ ions seriously. When α-NaYF_4_:5%Er^3+^ NCs calcined at 500 °C, just as B-NYF, A-NYF samples contain many inner defects and surface defects. The former is attributed to the phase transformation from cubic to hexagonal, and the latter is owing to volatilization of organic ligands. Besides, it can be found in FTIR pattern (Supplementary Fig. S11) and the insert in Fig. 7, the carbonization of organic ligands in as-prepared α-NaYF_4_:5%Er^3+^ NCs leads the products become black, which can efficiently absorb excitation and emission luminescence. Consequently, no MIR emission is detected in A-NYF-500 samples. Because of better crystallinity at higher temperature, A-NYF samples treated at 600 °C reveal stronger MIR emission than at 500 °C. Upon 600 °C, A-NYF samples agglomerate into nano-blocks with large size, which can reduce defects greatly. Furthermore, with an increase of temperature, A-NYF samples have more excellent crystallinity. As a result, an obvious enhancement of MIR fluorescence is observed along with the increase of temperature. When temperature reaches 1000 °C, emission peaks have a slight red shift, which may be due to the entrance of some O atoms into crystal structure in the way of replacing F atoms. It is surprising that, the same consequence is also found on A-NYF samples, which is when α phase as main phase, emission peak has an obvious splitting.

## Discussion

It is well known that, for MIR laser operation, host materials must be non-hygroscopic and characterized by low phonon energy, considering that there is a strong absorption at ~3 μm of H_2_O molecules, and increase of non-radiative relaxations by high phonon energies can reduce MIR emission remarkably. However, in our study, another two factors show more intense effect on MIR emission of Er^3+^ ions. The FTIR results of β-NaYF_4_:5%Er^3+^ NCs calcined at different temperatures (Supplementary Fig. S6) demonstrate there is no OH^−^ in the structure of B-NYF samples, but weaker MIR emission is detected in B-NYF-300 to −600 samples due to occurrence of defects produced by volatilization of organic ligands and phase transformation. With the increase of temperature, the significantly decreased defects and increased crystallinity result in the gradual enhancement of MIR emission of Er^3+^ ions in B-NYF samples. Similarly, A-NYF samples obtained at different temperatures also contain no OH^−^ based on its FTIR analysis (Supplementary Fig. S11), but the defects lead to weaker MIR emission in A-NYF-500 and A-NYF-600 samples. From 700 °C, A-NYF samples have an obvious enhancement of MIR emission, which results from the more excellent crystallinity. According to above results, it can be deduced that the defect and crystallinity have more significant influence on the MIR fluorescence of Er^3+^ ions in crystal hosts.

**Figure 8:**
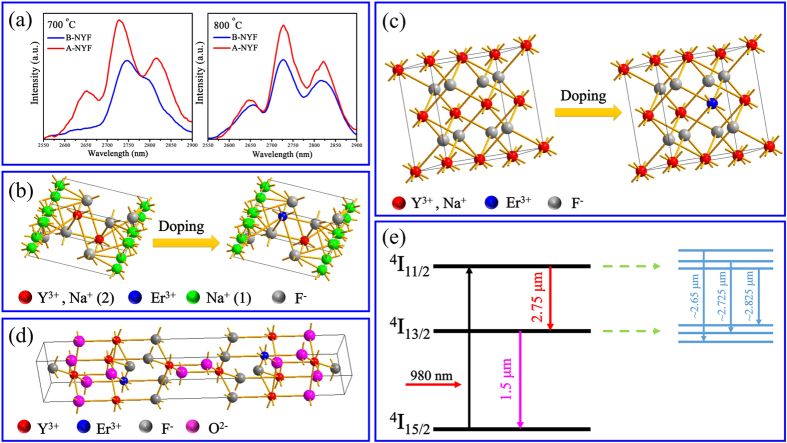
(**a**) The comparison of MIR emission spectra between B-NYF and A-NYF samples obtained at different temperatures. (**b**,**c**) Schematic presentation of hexagonal and cubic phase NaYF_4_ structures, respectively. (**d**) The crystal structure of the rhombohedral phase Er^3+^-doped YOF. (**e**) Energy levels of Er^3+^ ions and possible emission pathways of splitting energy levels.

Figure 8(a) shows the comparison of MIR emission between B-NYF and A-NYF samples treated at 700 °C and 800 °C. As one can see, A-NYF samples have stronger MIR emission intensity than B-NYF samples. Check their corresponding XRD results (Figs 2 and 6) carefully, more hexagonal phase can be detected in B-NYF samples, which can be neglected in A-NYF samples, leading to more defects and worse crystallinity for B-NYF samples. Based on above results, defect and crystallinity are the main influencing factors for MIR emission of Er^3+^ ions in our study, so B-NYF samples display weaker MIR emission. Moreover, it can be noted that NaYF_4_ samples with pure hexagonal phase or hexagonal phase as main phase, only have one MIR emission peak at ~2.75 μm, while the emission peak splits into three emission peaks along the appearance of cubic phase. Hence, we deduce Er^3+^ ions in cubic phase occur energy level splitting.

Schematic of hexagonal- and cubic-phase NaYF_4_ structures are presented in Fig. 8(b,c). In hexagonal phase NaYF_4_, an ordered array of F^−^ ions offers two types of cation sites: one occupied by Na^+^, and the other occupied by Y^3+^ and Na^+^, so Y^3+^ ions are located in nine-coordinated sites with low-symmetry. In contrast, in the crystal structure of cubic phase NaYF_4_ containing one type of high-symmetry cation site, fluorite structures (CaF_2_) are formed, with the cation sites randomly occupied by Na^+^ and Y^3+^, so Y^3+^ ions occupy the eight-coordinated cubic symmetry site^11^. When doping Er^3+^ ions into to the NaYF_4_ crystals, Er^3+^ ions will replace Y^3+^ ions and occupy its lattice sites, as displayed in Fig. 8(b,c). Therefore, in hexagonal phase, Er^3+^ ions are located in a low-symmetry nine-coordinated site, and in cubic phase, Er^3+^ ions reside in the eight-coordinated cubic polyhedra with high symmetry. For cubic symmetry, the energy levels of Er^3+^ ions can be decomposed into irreducible representations. It has been reported, when Er^3+^ ions doped into β-PbF_2_ crystal which can offer an eight-coordinated cubic symmetry, the ^*4*^*I*_*15/2*_ ground energy level and ^*4*^*I*_*13/2*_ excited energy level of Er^3+^ ions all split into multiplet^37^,^38^. Cubic phase NaYF_4_ crystal, owning similar structure with β-PbF_2_ crystal, also provides Er^3+^ ions an eight-coordinated cubic symmetry, leading to energy level splitting of Er^3+^ ions, so there are three MIR emission peaks detected in α-NaYF_4_:5%Er^3+^ crystals. For nine-coordinated site, its symmetry is too low to realize Er^3+^ ions multiplet emission, so there is only one MIR emission peak detected in hexagonal phase. In rhombohedral phase YOF crystals, Y^3+^ ions are coordinated by four oxide and four fluoride anions in a bicapped trigonal antiprism arrangement and all ions (F^−^, O^2−^, and Y^3+^) occupy the six-fold 6c Wyckoff positions with the same C_3ν_ site symmetry, as shown in Fig. 8(d) 34. Er^3+^ ions in obtained YOF crystals reside in eight-coordinated cubic symmetry, too. Thereby, three MIR splitting peaks were detected in B-NYF-1000 samples. The number of possible Stark levels of each energy is determined by the quantum number of the total angular momentum *J* of the energy level and the number of electrons of the considered ion^39^. Following Kramer’s theorem, each energy level splits into a maximum number of Stark levels^37^,^39^,^40^,^41^, with


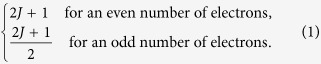


Each Er^3+^ ion contains 65 electrons, so that is to say there should be 6 sub-levels for ^*4*^*I*_*11/2*_ state and 7 sub-levels for ^*4*^*I*_*13/2*_ state theoretically. However, eight-coordinated cubic symmetry is a relatively lower symmetry. Furthermore, due to Er^3+^ (0.0881 nm) has smaller iron radius than Y^3+^ (0.0893 nm)^22^, the actual symmetry of Er^3+^ ions in these excited-states is slightly lower. In fact, Er^3+^ can’t achieve complete split when in eight-coordinated site of cubic phase NaYF_4_, and only three splitting peaks detected for α-NaYF_4_:5%Er^3+^ crystals. Thus, when Er^3+^ ions occupy cubic symmetry site in α-NaYF_4_ crystals, the ^*4*^*I*_*11/2*_ and ^*4*^*I*_*13/2*_ energy levels occur imperfect splitting, and realize MIR emission with three splitting peaks by radiative transition of electrons from sub-levels of ^*4*^*I*_*11/2*_ state to sub-levels of ^4^I_13/2_ state, as shown in Fig. 8(e). The specific study about level splitting of Er^3+^ in α-NaYF_4_ crystal still need further investigation.

In summary, monodisperse β-NaYF_4_:5%Er^3+^ nanorods and α-NaYF_4_:5%Er^3+^ nanospheres were synthesized by hydrothermal and co-precipitation method, respectively. For the first time, MIR emission attributed to radiative transition from ^*4*^*I*_*11/2*_ to ^*4*^*I*_*13/2*_ level of Er^3+^ ions was realized both in β- and α-NaYF_4_ NCs. Meanwhile, MIR fluorescence enhanced remarkably after calcination at high temperature. In comparison, A-NYF products with controllable morphology, smaller particle size, better uniformity and intense MIR emission present more possibility combining with glass to form NGC. During the calcination process, the phase transformation of NaYF_4_ between hexagonal and cubic phase was investigated in detail. The results show that as-prepared β-NaYF_4_ is located in stable state at low temperature, and transform into α-NaYF_4_ upon ~600 °C, while as-prepared α-NaYF_4_ transform into β-NaYF_4_ at low temperature, and then the β-NaYF_4_ transform back α-NaYF_4_ upon ~600 °C. Further increase of temperature will result in oxidation of NaYF_4_. Based on FTIR and PL spectra results, it can be deduced that the defect and crystallinity play a major role in MIR emission of Er^3+^ ions. Moreover, an interesting phenomenon attracts our attention, i.e. the MIR emission peak of Er^3+^ ions splits into three emission peaks in α-NaYF_4_ NCs while there is only emission peak in β-NaYF_4_ NCs, which is due to the difference of site symmetry in both cubic and hexagonal phases. Although as-prepared α-NaYF_4_:5%Er^3+^ NCs own spherical morphology and small size, bigger irregular nanoparticles are obtained on account of phase transformation after calcination, which will influence the compound properties of crystals. Hence, further investigation can be focused on suppressing phase transformation to control the morphology.

## Methods

### Preparation of β-NaYF_4_:Er^3+^ NCs

β-NaYF_4_ NCs doped with Er^3+^ ions were prepared using hydrothermal method based on oleic acid (OA 90 wt%, A.R.) as a stabilizing agent^16^. 1.4 g of NaOH (A.R.), 15 mL of OA, and 24.6 mL of C_2_H_5_OH (A.R.) were well mixed under stirring at room temperature to obtain a white viscous solution. 11.4 mL of 0.5 M Y(NO_3_)_3_ and 0.6 mL of 0.2 M Er(NO_3_)_3_ were added with vigorous stirring until a translucent solution was obtained. After 10 minutes of stirring, 12 mL of 1.0 M NaF solution was added dropwise into the above solution under vigorous stirring. After aging for 30 min, the mixture was transferred to a Teflon-lined 100 mL capacity autoclave, and heated at 200 °C for 12 h. When the autoclave was air-cooled down to room temperature, the samples were washed alternately by deionized water and ethanol, and dried at 80 °C for 8–12 h in air. Finally, the precursor powders were calcined at different temperatures for 2 h.

### Preparation of α-NaYF_4_:Er^3+^ NCs

Monodisperse α-NaYF_4_:Er^3+^ spheres were prepared according to the literature with some modification^42^. In a typical synthesis, 9.5 mL of 0.2 M Y(NO_3_)_3_ and 0.5 mL of 0.2 M Er(NO_3_)_3_ were mixed with an aqueous solution of ethylenediamine tetraacetic acid disodium salt (Na_2_EDTA, A.R.) under vigorous magnetic stirring to obtain a white complex. Afterwards, another solution of NaF (24 mmol added into 30 mL deionized water) was added into the above white solution and stirred for 1 h at room temperature. The as-obtained precipitates were centrifuged, washed several times with deionized water and ethanol, and then dried at 80 °C for 8–12 h in air. Finally, the precursor powders were calcined at different temperatures for 2 h.

### Characterization

The crystalline structure of β-NaYF_4_:Er^3+^ and α-NaYF_4_:Er^3+^ NCs were investigated by X-ray diffraction (XRD) (Bruker, D8 ADVANCE analysis with Cu Kα radiation operated at 40 kV and 40 mA, λ = 0.15418 nm, scanning step 0.02°, scanning speed 0.1 s per step). The morphology and size distribution of NCs were observed by field emission-scanning electron microscopy (FE-SEM, Nova NanoSEM403, FEI, Netherlands), and high-resolution transmission electron microscopy (HR-TEM, 2100F, JEOL, Japan) equipped with an energy-dispersive X-ray spectrometer (EDS). Thermal analysis of the precursor powders were detected by simultaneous thermal analyzer (STA, STA449C/3/MFC/GJUPITEY, NETZSCH, Germany). A heating rate of 10 °C/min was adopted. The Raman spectra were measured using a Raman spectrometer (Renishaw in Via, Gloucestershire, UK) with a 532 nm laser as the excitation source. The organic groups in NCs were detected from Fourier transform-infrared spectroscopy (FT-IR, Vector-33, Bruker, Switzerland). The photoluminescence spectra were measured on a high resolution spectrofluorometer (Edinburgh Instruments FLS 920), which were excited by a 976 nm laser diode (LD).

## Additional Information

**How to cite this article**: Yang, D. *et al.* Controllable Phase Transformation and Mid-infrared Emission from Er^3+^-Doped Hexagonal-/Cubic-NaYF_4_ Nanocrystals. *Sci. Rep.* **6**, 29871; doi: 10.1038/srep29871 (2016).

## Supplementary Material

Supplementary Information
