# Supplementary material for: Controllable Phase Transformation and Mid-infrared Emission from Er3+-Doped Hexagonal-/Cubic-NaYF4 Nanocrystals
Source: Sci Rep. 2016 Jul 25;6:29871. doi: 10.1038/srep29871 (PMC4958980; doi:10.1038/srep29871)
Supplement: Supplementary Information [file srep29871-s1.pdf]

Supporting information

Controllable Phase Transformation and Mid-infrared Emission from Er^{3+}-Doped Hexagonal-/Cubic-$NaYF_4$ Nanocrystals

Dandan Yang[1], Dongdan Chen[1], Huilin He[1], Qiwen Pan[1], Quanlan Xiao[2], Jianrong Qiu[1], and Guoping Dong[1]*

[1]State Key Laboratory of Luminescent Materials and Devices and Institute of Optical Communication Materials, School of Materials Science and Engineering, South China University of Technology, Guangzhou 510640, China

[2]SZU-NUS Collaborative Innovation Center for Optoelectronic Science & Technology, Key Laboratory of Optoelectronic Devices and Systems of Ministry of Education and Guangdong Province, College of Optoelectronic Engineering, Shenzhen University, Shenzhen 518060, China

*Correspondence to [dgp@scut.edu.cn (G. Dong)]

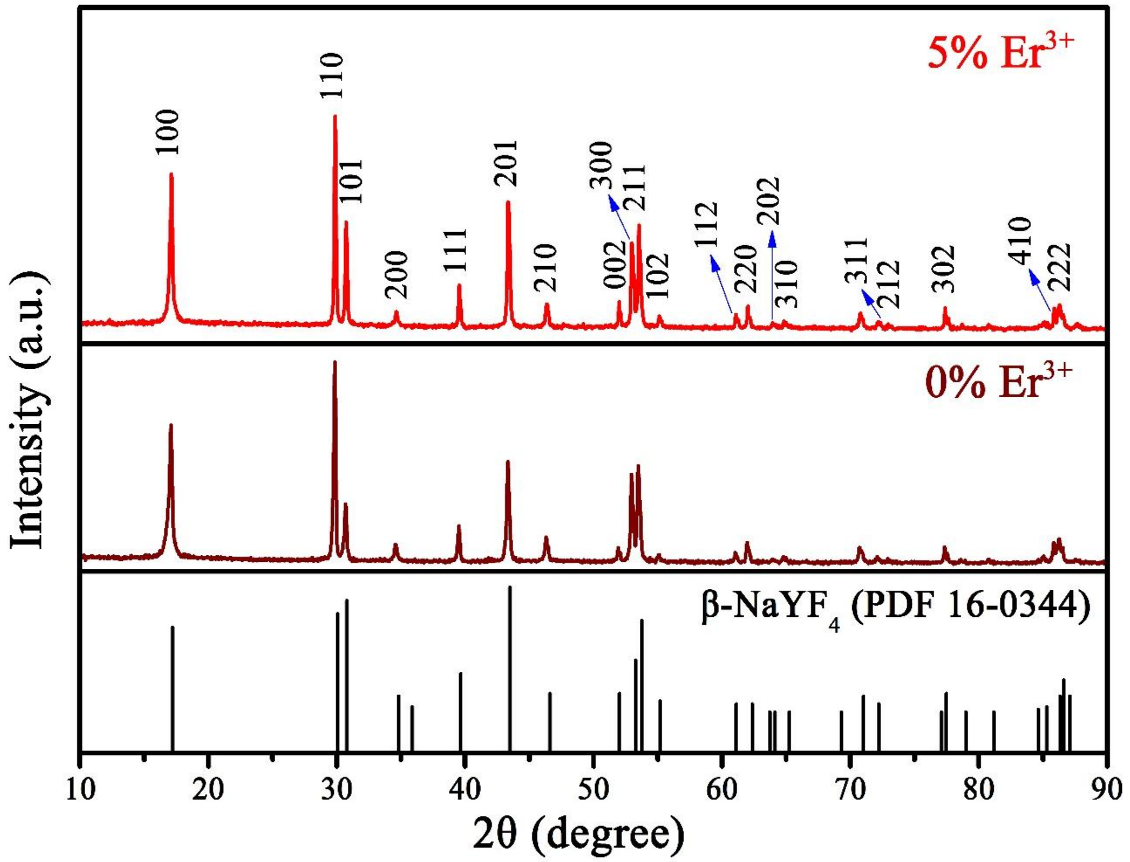

Figure. S1 XRD patterns of the as-prepared β-$NaYF_4$ and β-$NaYF_4$:5%Er^{3+} nanocrystals.

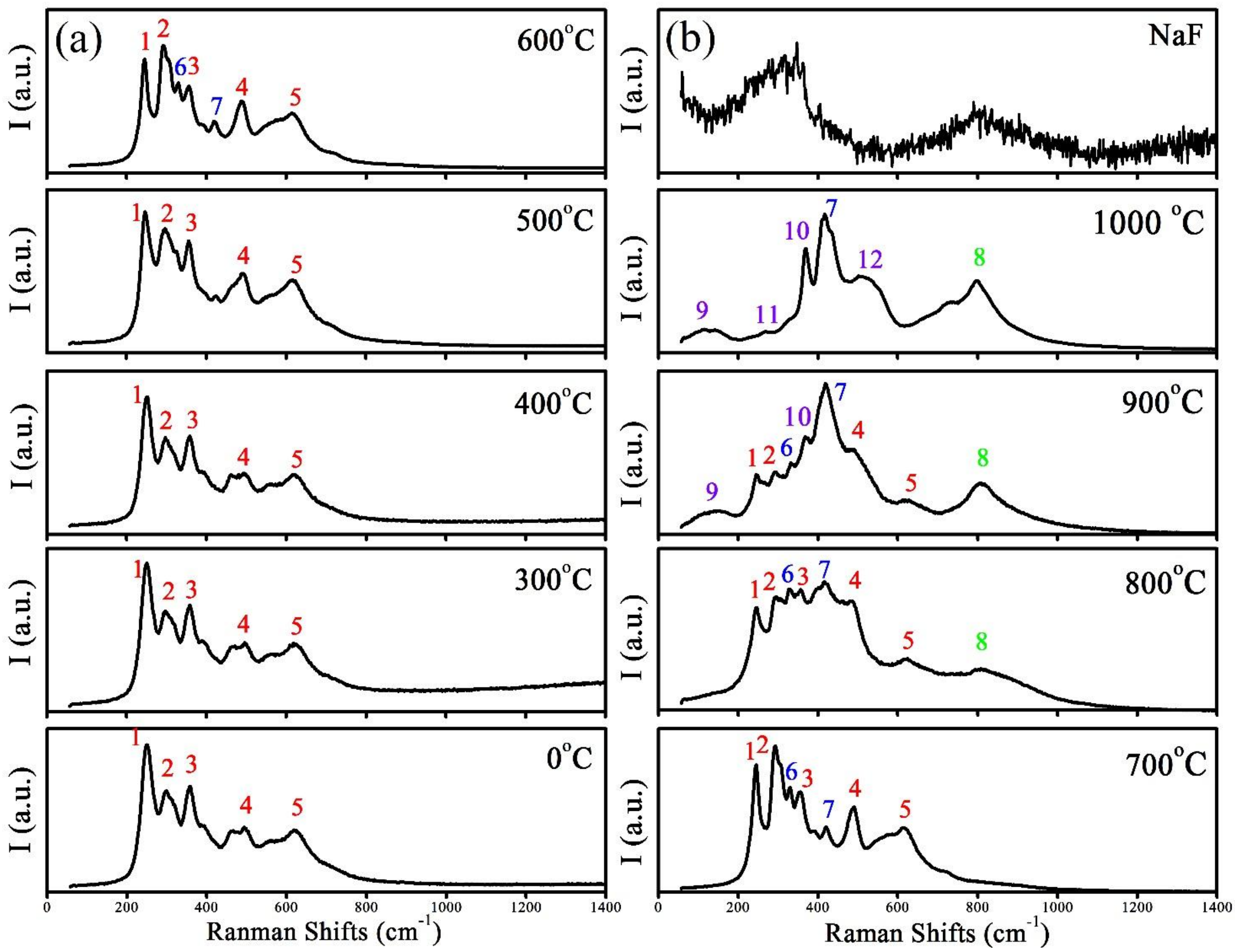

Figure. S2 Raman spectra of β-$NaYF_4$:5%Er^{3+} nanocrystals calcined at different temperatures for 2 h: (a) 0-600°C; (b) 700-1000°C and NaF. The 0°C stands for as-prepared β-$NaYF_4$:5%Er^{3+} nanocrystals. In this spectra, the dominant peaks 1-5 at 248 cm^{-1}, 299 cm^{-1}, 358 cm^{-1}, 460 cm^{-1}, and 618 cm^{-1} are assigned to those of β-$NaYF_4$[1,2], while the other two weak peaks near 4 and 5 are attributed to the vibrations from the organic surfactants[3]. The vibrations from the α-$NaYF_4$ can be seen at 329 cm^{-1} (6) and 419 cm^{-1} (7)[4], while peaks 9-12 at 140 cm^{-1}, 370 cm^{-1}, 270 cm^{-1} and 501 cm^{-1} are owing to the vibrations from YOF[5]. In contrast to Raman synthesis of NaF raw material, the peak 8 at 799 cm^{-1} is the vibration from NaF.

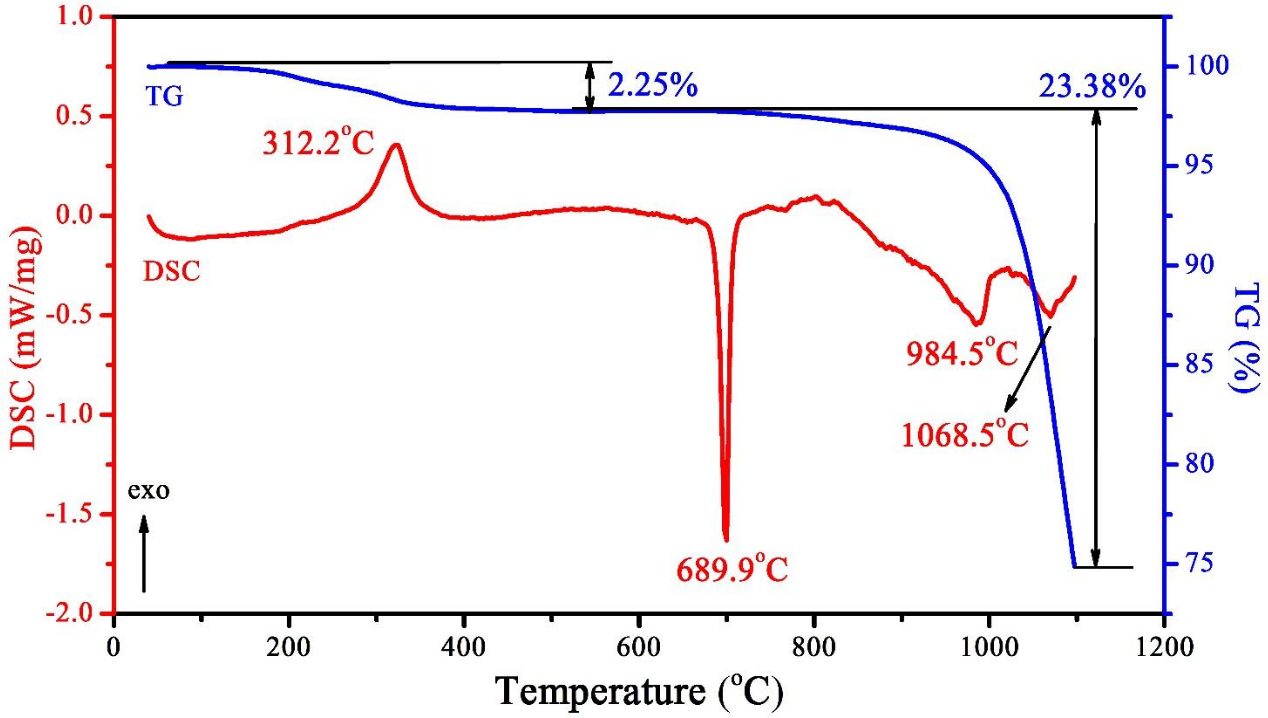

Figure. S3 TG-DSC curves of β-$NaYF_4$:5%Er^{3+} nanocrystals in air atmosphere with a heating rate of 10°C/min. The exothermic peak at around 312.2°C owing to the dehydration and vaporization of various fluoride by-products and organic ligands on the nanorods surface. This is also the reason for the 2.25% weight lost during this process. The intense endothermic peak at 689.9°C is due to the phase transformation from hexagonal to cubic phase. The endothermic peak at 984°C is relative to another phase transformation from $NaYF_4$ to YOF crystals. Meanwhile, the weight lost about 23.38% here is because the oxygen atoms have replaced fluoride atoms to join in YOF crystal lattice. The last endothermic peak at 1058.5°C is probably owing to a few YOF were further oxidized to Y_2O_3 [6].

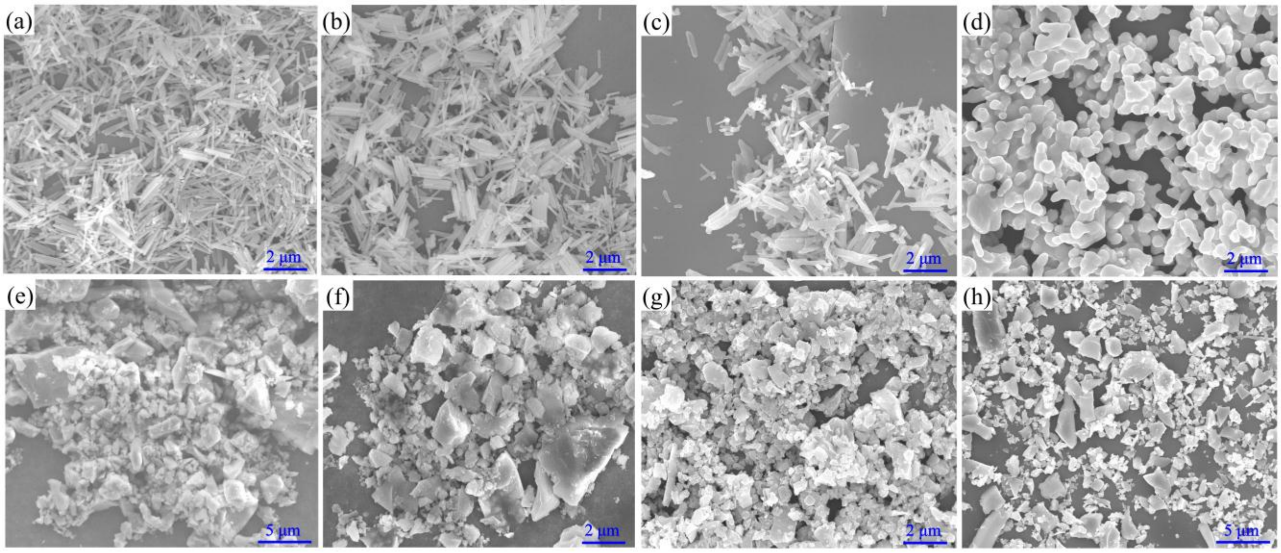

Figure. S4 SEM images of β-$NaYF_4$:5%Er^{3+} nanocrystals calcined at different temperatures for 2h: (a) 300°C; (b) 400°C; (c) 500°C; (d) 600°C; (e) 700°C; (f) 800°C; (g) 900°C; (h) 1000°C.

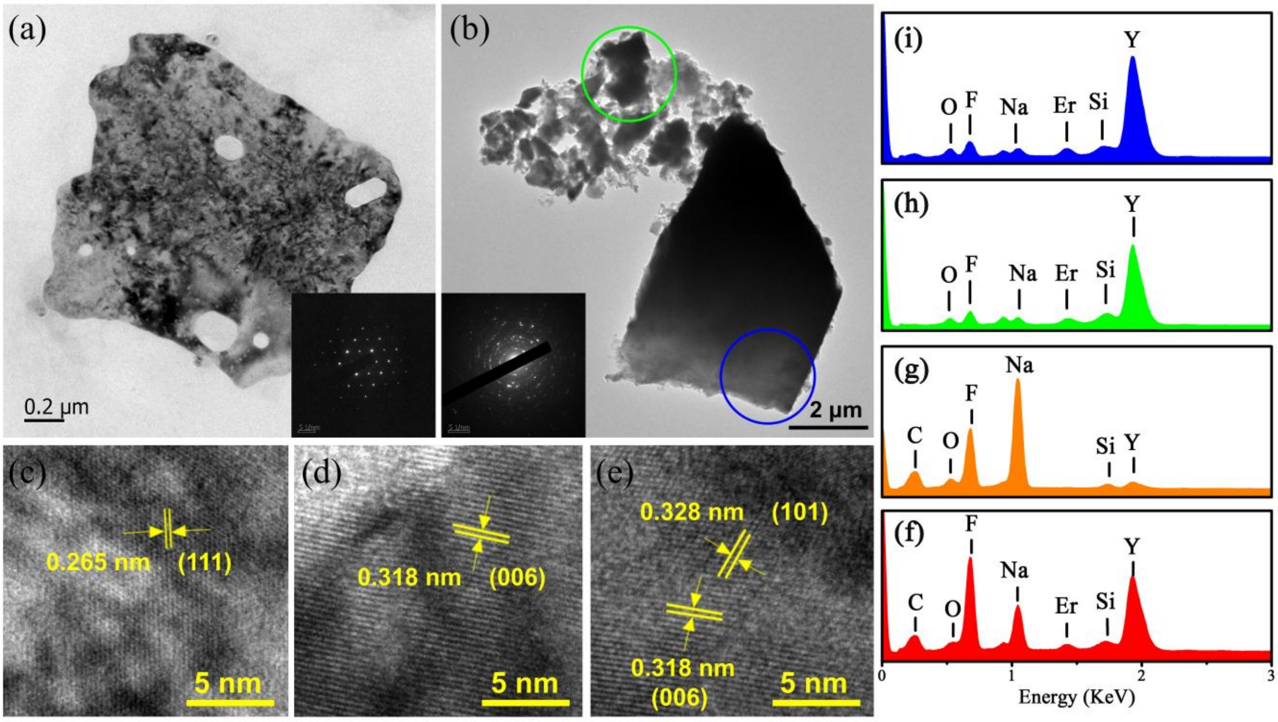

Figure. S5 (a) and (b) TEM images of β-$NaYF_4$:5%Er^{3+} nanocrystals calcined at 1000°C for 2 h. (c-e) HRTEM images: (c) samples in (a), (d) green circle part in (b), and (e) blue circle part in (b). (f-i) EDS spectra: (f) as-prepared β-$NaYF_4$:5%Er^{3+} nanocrystals, (g) samples in (a), (h) green circle part, and (i) blue circle part in (b), respectively. Inserts in (a) and (b) are SAED patterns of samples in (a), green circle part in (b), respectively.

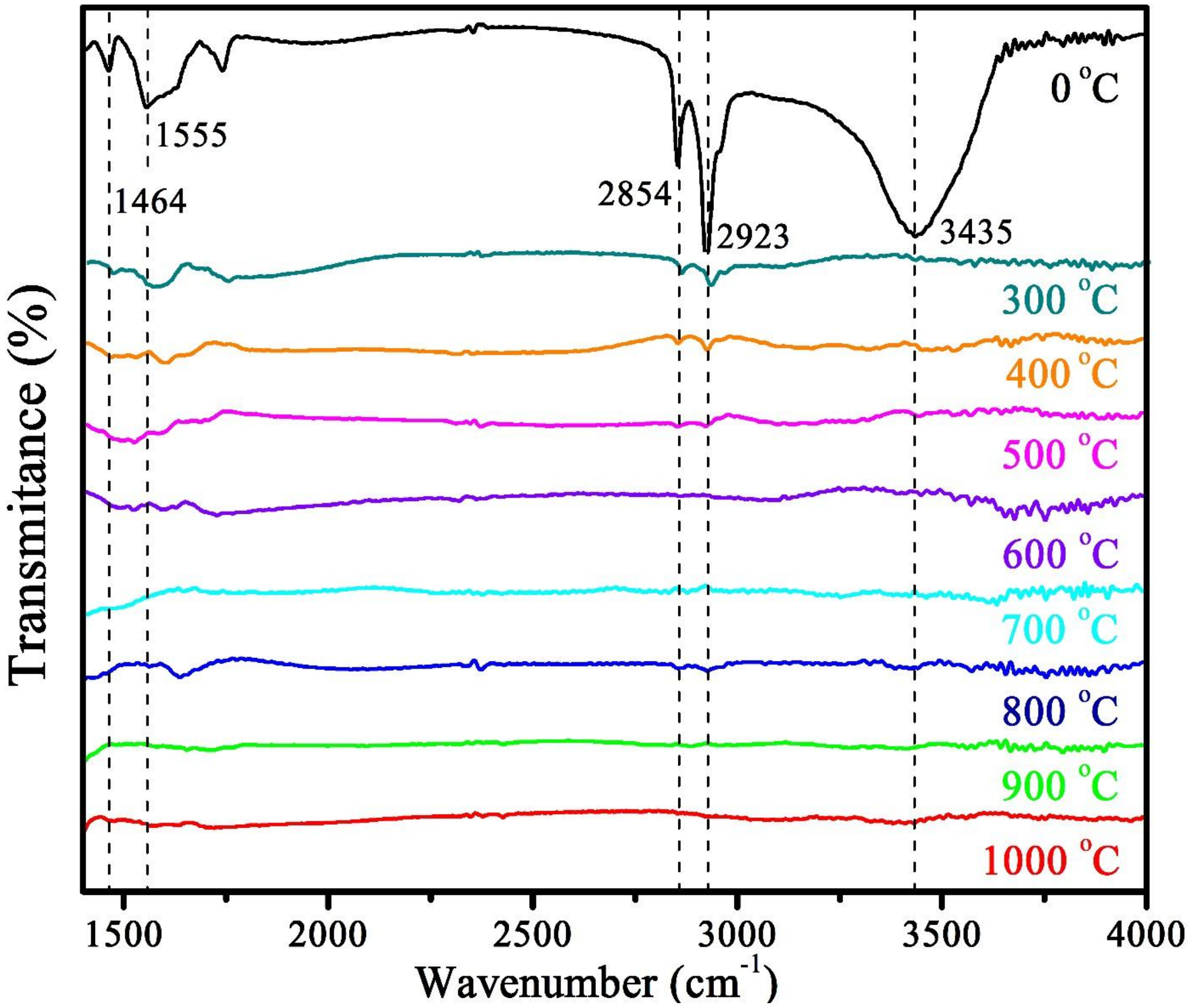

Figure. S6 FTIR spectra of β-$NaYF_4$:5%Er^{3+} nanocrystals calcined at different temperatures for 2 h. The 0°C stands for as-prepared β-$NaYF_4$:5%Er^{3+} nanocrystals. In the picture, the band at ~3435 cm^{-1} stands for the stretching vibration of the OH^- group[7]. The two weak bands at ~1555 and ~1464 cm^{-1} stand for the asymmetric (ν_{as}) and symmetric (ν_s) stretching vibration of carbonyl (C=O) group in OA molecule, while the bands at ~2923 and ~2584 cm^{-1} stand for the asymmetric (ν_{as}) and symmetric (ν_s) stretching vibration of methylene (-CH_2-) group, respectively, which is also from OA molecule[7].

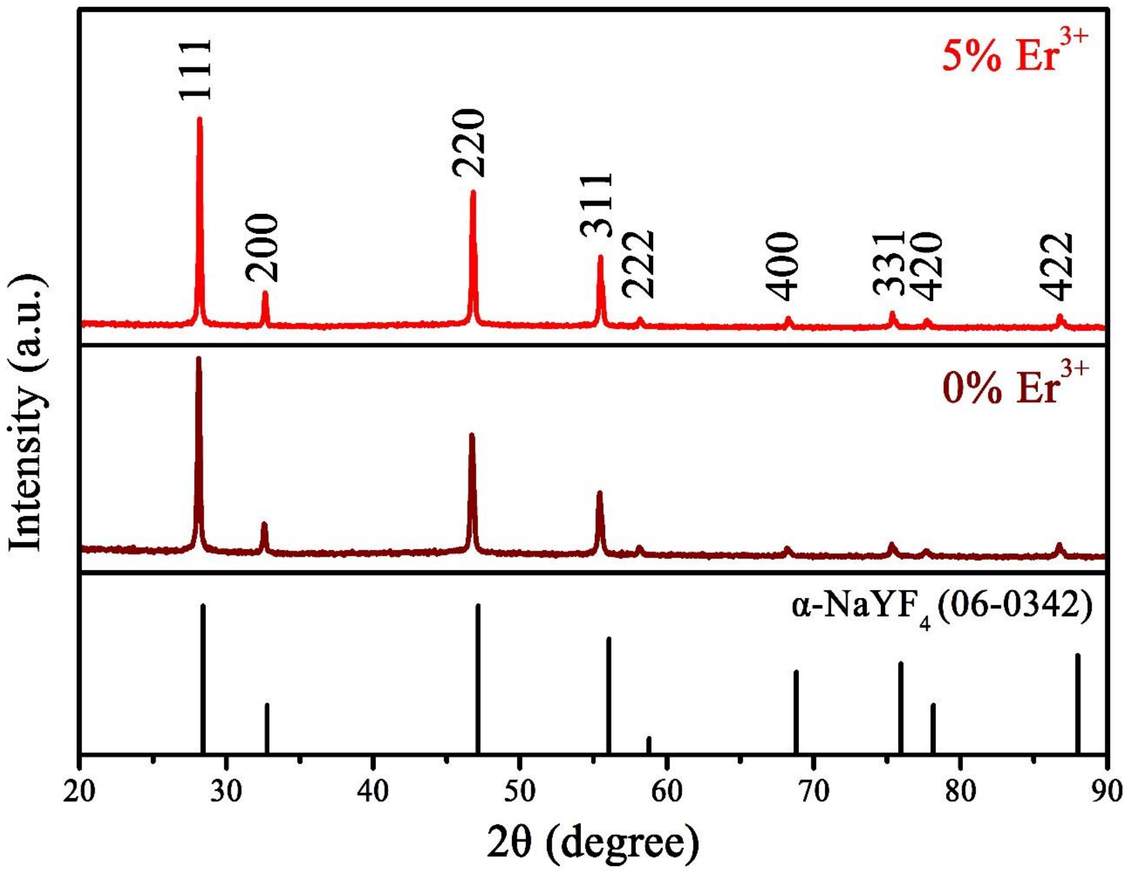

Figure. S7 XRD patterns of the as-prepared α-$NaYF_4$ and α-$NaYF_4$:5%Er^{3+} nanocrystals.

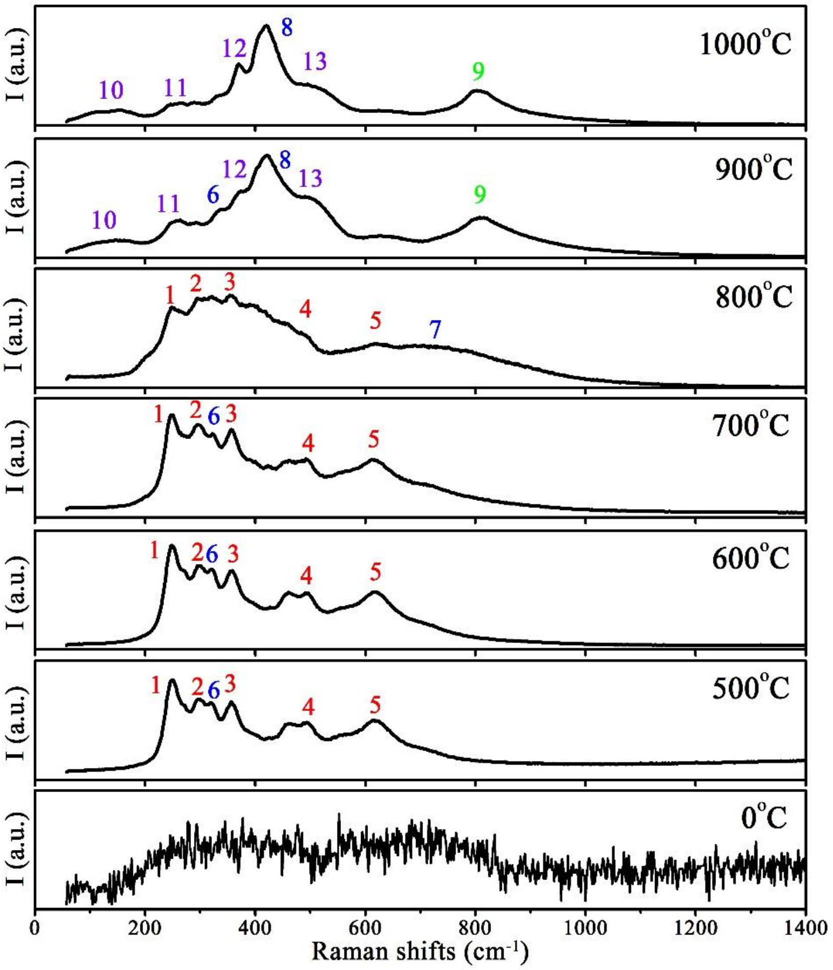

Figure. S8 Raman spectra of α-$NaYF_4$:5%Er^{3+} nanocrystals calcined at different temperatures for 2 h. The 0°C stands for as-prepared α-$NaYF_4$:5%Er^{3+} nanocrystals. In this spectra, 1-5 Raman peaks are assigned to the vibration from β-$NaYF_4$[1,2], 6-8 Raman peaks are related to the vibration from α-$NaYF_4$[4], 10-13 Raman peaks are ascribed to the vibration from YOF[5], and the 9 Raman peak represents NaF.

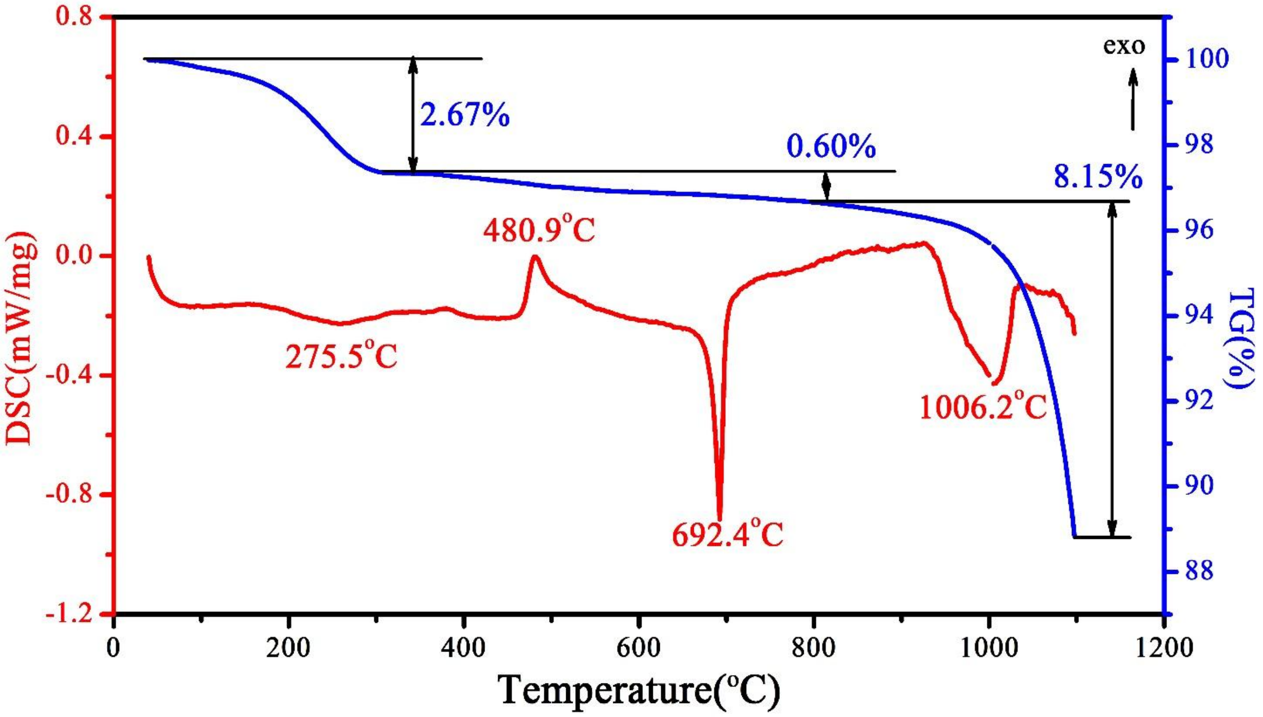

Figure. S9 TG-DSC curves of α-$NaYF_4$:5%Er^{3+} nanocrystals in air atmosphere with a heating rate of 10°C/min. The first endothermic peck at 275.5°C is due to dehydration and combustion of organic ligands, resulting in 2.67% loss of weight. The exothermic peak at 480.9°C is attributed to cubic phase transforming into hexagonal phase. The vaporization of some residual organic ligands leads to 0.6% loss of weight during this process. The other two endothermic peaks at 692.4°C and 1006.2°C are due to phase transformation of β→α and formation of YOF. The weight loss during the latter endothermic process is because some O atoms have replaced F atoms.

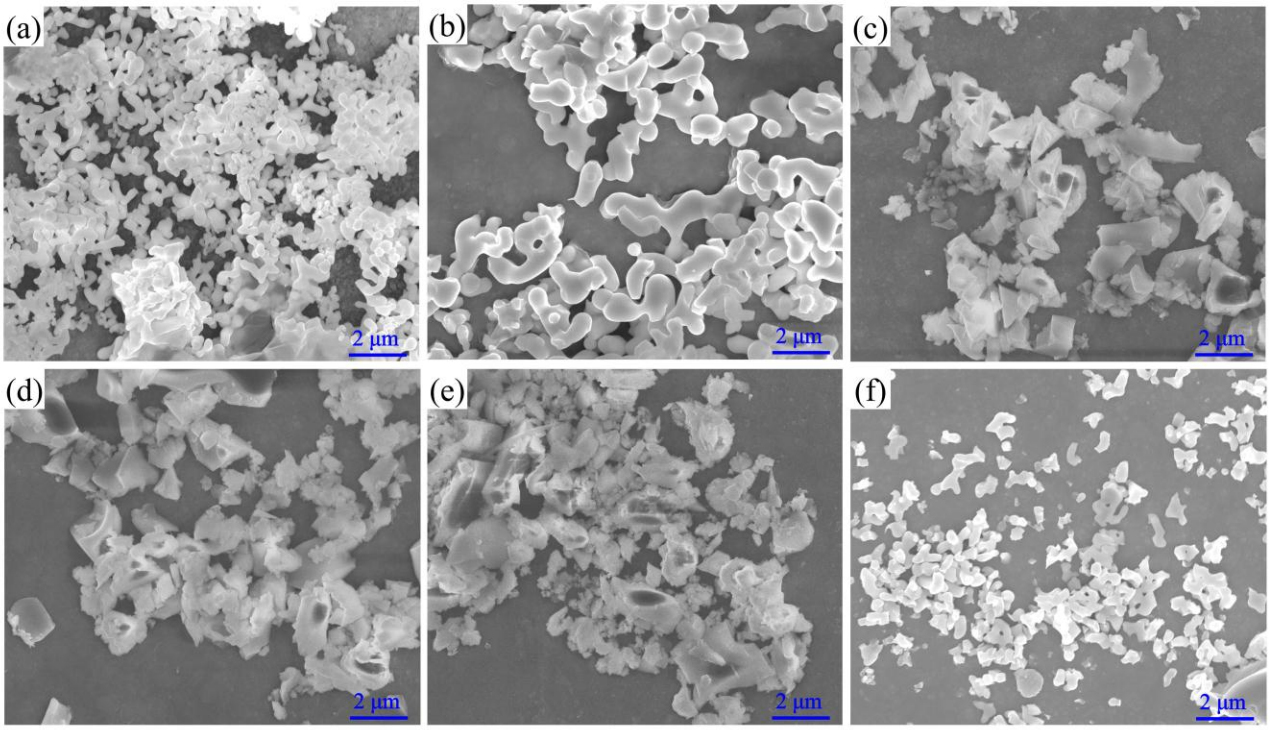

Figure. S10 SEM images of α-$NaYF_4$:5%Er^{3+} nanocrystals calcined at different temperatures for 2 h: (a) 500°C; (b) 600°C; (c) 700°C; (d) 800°C; (e) 900°C; (f) 1000°C.

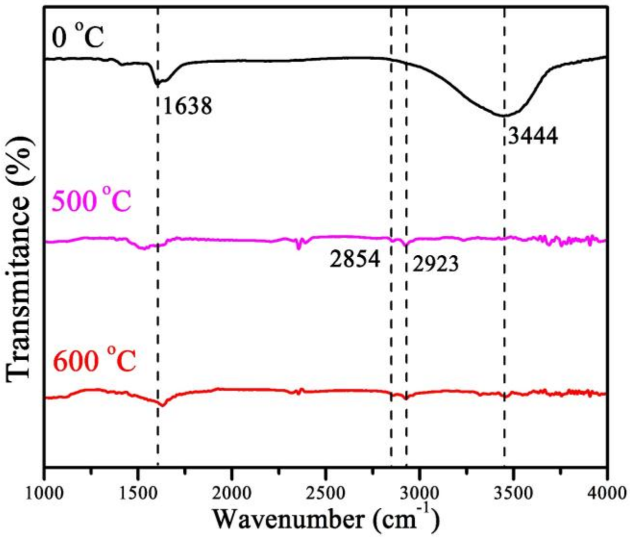

Figure. S11 FTIR spectra of α-$NaYF_4$: 5%Er^{3+} nanocrystals calcined at different temperatures for 2 h. The 0°C stands for the as-prepared α-$NaYF_4$:5%Er^{3+} nanocrystals. In the picture, the band at 1638 cm^{-1} stands for the bending vibration of the N-H bond from EDTA[8]. The bands at ~2923 and ~2584 cm^{-1} stand for the asymmetric (ν_{as}) and symmetric (ν_s) stretching vibration of methylene (-CH_2-) group, respectively[6]. The band at ~3435 cm^{-1} stands for the stretching vibration of the OH^- group[6].

References

1. Wang, G. *et al.* Controlled synthesis and luminescence properties from cubic to hexagonal $NaYF_4:Ln^{3+}$ (Ln= Eu and Yb/Tm) microcrystals. *J. Alloy. Compd.* **475**, 452-455 (2009).

2. Renero-Lecuna, C. *et al.* Origin of the high upconversion green luminescence efficiency in β-$NaYF_4$: 2%Er^{3+}, 20%Yb^{3+}. *Chem. Mater.* **23**, 3442-3448 (2011).

3. Luo, X. & Akimoto, K. Upconversion properties in hexagonal-phase $NaYF_4:Er^{3+}/NaYF_4$ nanocrystals by off-resonant excitation. *Applied Surface Science* **273**, 257-260 (2013).

4. Assaaoudi, H., Shan, G.-B., Dyck, N. & Demopoulos, G. P. Annealing-induced ultra-efficient NIR-to-VIS upconversion of nano-/micro-scale α and β $NaYF_4:Er^{3+}$, Yb^{3+} crystals. *CrystEngComm* **15**, 4739-4746 (2013).

5. Zhang, Y. *et al.* YOF nano/micro-crystals: morphology controlled hydrothermal synthesis and luminescence properties. *CrystEngComm* **16**, 2196-2204 (2014).

6. Chai, G., Dong, G., Qiu, J., Zhang, Q. & Yang, Z. Phase transformation and intense 2.7 μm emission from Er^{3+} doped YF_3/YOF submicron-crystals. *Sci. Rep.* **3** (2013).

7. Ding, M. Y. *et al.* Effect of heat-treatment temperature on upconversion luminescence of beta-$NaYF_4:Yb^{3+}$, Er^{3+} nano/microparticles. *J. Inorg. Mater.* **28**, 146-152 (2013).

8. Chen, J. *et al.* Controllable synthesis of $NaYF_4$:Yb, Er upconversion nanophosphors and their application to in vivo imaging of Caenorhabditis elegans. *J. Mater. Chem.* **21**, 2632-2638 (2011).
